# Neuropeptide FF receptor 2 inhibits capsaicin-induced CGRP Upregulation in mouse trigeminal ganglion

**DOI:** 10.1186/s10194-020-01152-z

**Published:** 2020-07-08

**Authors:** Ya-Tin Lin, Zachary Yu, Sze-Chi Tsai, Po-Hung Hsu, Jin-Chung Chen

**Affiliations:** 1 Graduate Institute of Biomedical Sciences, Department of Physiology and Pharmacology, Chang Gung University, 259 Wenhua 1st Road, Guishan Dist, Taoyuan City, 33302 Taiwan; 2 Healthy Aging Research Center, Chang Gung University, 259 Wenhua 1st Road, Guishan Dist, Taoyuan City, 33302 Taiwan; 3 Department of Medicine, Chang Gung University, 259 Wenhua 1st Road, Guishan Dist, Taoyuan City, 33302 Taiwan; 4 Department of Biomedical Sciences, Chang Gung University, 259 Wenhua 1st Road, Guishan Dist, Taoyuan City, 33302 Taiwan; 5 Center for Advanced Molecular Imaging and Translation, Chang Gung Memorial Hospital, 5 Fu-Hsing Street. Guishan Dist, Taoyuan City, 33302 Taiwan; 6 Department of Electrical Engineering, Chang Gung University, 259 Wenhua 1st Road, Guishan Dist, Taoyuan City, 33302 Taiwan; 7 Neuroscience Research Center, Chang Gung Memorial Hospital, 5 Fu-Hsing Street. Guishan Dist, Taoyuan City, 33302 Taiwan

**Keywords:** Trigeminovascular pathway, CGRP, NPFFR2, Neuropeptide FF (NPFF), Trigeminal ganglion, Headache, Migraine, Capsaicin

## Abstract

**Background:**

Stimulation of trigeminovascular pathway is widely used to establish the headache animal model. Headache is a common neurological disorder, in which symptomatic attacks are mediated by calcitonin-gene-related peptide (CGRP). CGRP is synthesized and released from the trigeminal ganglion to transmit pain signals under stimulation. On the other hand, Neuropeptide FF (NPFF) is a candidate transmitter/modulator for migraine, and stimulation of its receptor, NPFFR2, increases the expression and release of CGRP in mice sensory neurons. Here, we investigate the impact of NPFFR2 on trigeminal CGRP level in a capsaicin-induced headache mouse model.

**Methods:**

Mice were intracisternally injected with capsaicin into the cisterna magna to activate the trigeminovascular pathway and induce headache symptoms. Mice pretreated with *Npffr2*-shRNA or NPFFR2 knockouts were adopted to test the impact of NPFFR2 on capsaicin-induced CGRP upregulation in trigeminal ganglion. The gene silencing effect of *Npffr2*-shRNA in trigeminal ganglion was confirmed by real-time PCR. Trigeminal CGRP level was determined by immunofluorescence staining, and the percentage of CGRP-positive cell was calculated after setting the signal intensity threshold by Image J software. Amount of trigeminal CGRP in NPFFR2 overexpressed mice was also measured by CGRP ELISA.

**Findings:**

Infusion of capsaicin into the cisterna magna upregulated the CGRP in trigeminal ganglion and induced spontaneous pain behaviors including the reduction of locomotor activity and the increase of freezing behavior. Intracisternal injection of *Npffr2*-shRNA reduced the mRNA of *Npffr2* in trigeminal ganglion. Mice pretreatment with *Npffr2*-shRNA prevented capsaicin-induced CGRP upregulation in trigeminal ganglion. Similarly, CGRP upregulation was also reduced in NPFFR2 knockout mice. On the contrary, trigeminal CGRP was increased in NPFFR2 overexpressed mice.

**Conclusions:**

Reducing the level of NPFFR2 leads to the downregulation of capsaicin-induced CGRP in trigeminal ganglion, which would consequently attenuate the activation of trigeminovascular pathway. Thus, NPFFR2 could serve as a potential target for neuromodulation of cephalic pain.

## Introduction

The cause of the headache is linked to activation of the trigeminovascular pathway [1]. In this pathway, afferent nerves transmit nociceptive signals from the meninges and other peripheral areas to the trigeminal ganglion and trigeminal nucleus caudalis. The trigeminal ganglion amplifies pain through circuits involving calcitonin-gene-related peptide (CGRP) [2], which is synthesized in the trigeminal ganglion and released into cranial venous during migraine attacks [1]. Migraine is a common neurological disorder, in which patients normally experience acute attacks of unilateral headache with or without aura. In addition to headache, migraine attacks may also involve nausea, photophobia, phonophobia and cognitive dysfunction. CGRP has become a valuable target for headache therapies because its role to initiate the nociceptive transmission and responsible for the migraine attack [1–3].

Neuropeptide FF (NPFF) has recently been identified in a genome-wide association study as a candidate risk factor for migraine, with the top migraine-associated single nucleotide polymorphism in the *NPFF* gene locus (12q13.13) being rs11170566 [4]. Interestingly, NPFF acts through its receptor, NPFFR2, to increase the synthesis and release of CGRP from sensory neurons, which might be important in the regulation of headache symptoms [5].

The stimulation of trigeminovascular pathway is widely used to establish the headache animal model. Comparable to the inflammatory soup, capsaicin infusion into the cisterna magna can also stimulate the meningeal sensory pathway and activate the trigeminal nucleus caudalis [6, 7]. In this study, we evaluated the requirement of NPFFR2 for CGRP upregulation in a capsaicin-induced pain mouse model through the trigeminovascular activation. NPFFR2 was diminished by pretreatment with *Npffr2*-shRNA or eliminated in NPFFR2 knockout (KO) mice. The trigeminal CGRP in NPFFR2 overexpressed transgenic (Tg) mice were also measured.

## Materials and methods

### Animals

Male C57BL/6 wild-type (WT) mice (8 weeks old, 24–26 g) were purchased from the National Laboratory Animal Center (Taipei, Taiwan). NPFFR2 KO mice, NPFFR2 Tg mice and corresponding WT controls were bred in an SPF environment of Chang Gung University (AAALAC accreditation). Animals were randomly housed four to five per cage at 22 ± 1 °C, 50 ± 5% humidity, and 12 h light/dark cycle (lights on, 07:00). Food and water were available ad libitum. Animal handling and drug treatments were performed in strict accordance with the NIH Guide for the Care and Use of Laboratory Animals, and approved by the IACUC at Chang Gung University (CGU 14–014).

### Intra-olfactory bulb *Npffr2*-shRNA delivery

Five lentivirus (LV)-packaged mouse *Npffr2*-shRNA plasmids were purchased from the National RNAi Core Facility at Academia Sinica, Taiwan. LacZ-shRNA served as a control. Table 1 shows shRNA IDs and sequences. Olfactory bulb was chosen to test the silencing effect of individual shRNA due to its high expression of NPFFR2. Mice were anesthetized by intraperitoneal injection of ketamine (67 mg/kg) and xylazine (34 mg/kg), then fixed into a stereotaxic instrument (David Kopf Instruments, Tujunga, CA). *Npffr2* LV-shRNA (*Npffr2*-a-e) was injected into the bilateral olfactory bulb. Control and *Npffr2* LV-shRNA particles (3–5 × 10^9^ RIU/ml) were delivered at 1 μl/min for 1 min using a micro-syringe pump connected to PE-10 tubing with blunt tip of 30 gauge needle; the needle was kept in place for 5 min to prevent backflow. The coordinates for olfactory bulb were AP, + 3.92 mm; ML, ±1.0 mm and DV, − 1.4 mm from bregma (Paxinos and Franklin, 2001). One week after injection, gene silencing in the olfactory bulb was evaluated.

**Table 1:** Sequence for shRNA and real-time PCR primers

Name	ID	Sequence
*Npffr2-a* shRNA	TRCN0000027462	GCCTATCACATTGCTGGACAA
*Npffr2-b* shRNA	TRCN0000027446	GCGTATCATCAACATCTACAT
*Npffr2-c* shRNA	TRCN0000027471	GCGAAACGCAACATAGTCATA
*Npffr2-d* shRNA	TRCN0000027453	CCATCTGCAATAATGTTACAT
*Npffr2-e* shRNA	TRCN0000027484	GCATCACTGGTATTCAGATAT
Control shRNA	TRCN0000072232	CGTCGTATTACAACGTCGTGA
*Rpl35a*-forward		GCTGTGGTCCAAGGCCATTTT
*Rpl35a*-reverse		CCGAGTTACTTTTCCCCAGATGAC
*Npffr2*-forward		ACATCTACCCTTTCGCCCAC
*Npffr2*-reverse		GCTTCTCCCATTTCCTCTATCAA

### Real-time PCR

Total RNA was isolated using TRIzol® reagent (Invitrogen, Carlsbad, CA, USA). cDNA was made using EpiScript reverse transcriptase (Epicentre, WI, USA). Expression levels were measured by real-time PCR using SYBR and CFX real-time PCR detection system (Bio-Rad, Hercules, CA, USA). The PCR protocol was: 95 °C, 10 min, followed by 95 °C, 15 s and 60 °C, 30 s for 40 cycles. *Rpl35a* served as an internal control. Table 1 shows primer sequences.

### Intracisternal *Npffr2* LV-shRNA and capsaicin delivery

*Npffr2* LV-shRNA and capsaicin were delivered into the cisterna magna through intracisternal injection at designated time points. After anesthetizing mice, a surgical opening was made between the scalp and C1 spinal cord. A PE-10 catheter with 30 gauge needle filled with control or *Npffr2*-a LV*-*shRNA was inserted 2 mm into the cisterna magna. Mice were kept prone for 15 min before infusion of *Npffr2* LV-shRNA or control LV-shRNA (10 μl, 3–5 × 10^8^ RIU/ml) into the cisterna magna (2 μl/min, 5 min) using a CMA Syringe Pump (Harvard, MA, USA). The catheter was removed 10 min after injection. After surgery, mice were intraperitoneal injected with 200 μl saline, glucose (0.4 g/kg) and ampicillin (4 mg/kg) and allowed to recover under a heat source. One week after LV-shRNA injection, mice were anaesthetized again for capsaicin infusion or collected the trigeminal ganglion for analyzing the *Npffr2* gene knockdown. For capsaicin infusion, the needle was inserted in the cisterna magna and left for 1 h before the infusion. Capsaicin (1 nmole in 10 μl) or saline was infused into the cisterna magna (2 μl/min) for 5 min. After treatment, mice remained anaesthetized for 2 h. During the first 30 min, mice were placed in a reverse Trendelenburg position (− 30°). Afterward, mice were placed in prone position for 90 min before sacrificing for immunofluorescence staining. Capsaicin (Sigma-Aldrich, St. Louis, MO, USA) were dissolved in solution (saline: ethanol: Tween 80 = 8:1:1) as a 10 mM stock and further diluted with saline into 100 μM before intracisternal injection.

### Behavioral tests

WT mice injected with capsaicin were subjected to locomotor activity and freezing behavior measurements. Mice were intracisternally injected with saline or capsaicin under inhaled anesthesia of 1.5% of isoflurance, and placed in a reverse Trendelenburg position for 30 min. Mice were then awaked and waited for another hour prior to the behavioral tests. The locomotor activity was monitored in an open field arena (40 cm × 40 cm), quantified by tracking the amount of body movement for 30 min (EthoVision, Noldus, Wageningen, The Netherlands). Freezing behavior was defined as the change of surface area below 2% of the tracked mice body. The results are presented as the distance that mice moved (cm) or, duration of freezing behavior (sec) in every 3 min for a total session of 30 min.

### Immunofluorescence staining

The procedure was similar to previous reports [5]. The bilateral trigeminal ganglion was collected and sliced into 20-μm sections. The antibodies used were anti-CGRP (EMD Millipore Corp, Billerica, MA, USA) and Cy3-conjugated secondary antibody (Jackson ImmunoResearch, West Grove, PA, USA). Eight slides from unilateral trigeminal ganglion were obtained with a 120-μm interval sequentially and the range covered the whole ganglion. Sixteen slides from bilateral trigeminal ganglion were stained and the CGRP-positive cell numbers and total cell numbers were quantified after setting the signal intensity threshold in Image J software (NIH, Bethesda, MD, USA). The results were presented as the percentage of CGRP-positive cells (CGRP-positive cell numbers / total cell numbers × 100%).

### CGRP ELISA

Bilateral trigeminal ganglion was collected and sonicated in an ice-cold PBS solution. The homogenates were then centrifuged at 5000×*g* for 5 min at 4 °C. The supernatants were analyzed immediately according to the manufacturer’s protocols by CGRP ELISA kit (Cayman, Ann Arbor, Michigan, USA). The protein concentrations were determined from the pellets by Coomassie blue method with bovine serum albumin as standards. The concentration of CGRP measured by ELISA was calibrated with protein levels of the tissue pellets and presented as ng/mg protein.

### Statistical analysis

All data are expressed as mean ± standard error mean (SEM). Statistical analyses were performed using Prism7 (GraphPad, San Diego, USA). Silencing effect of *Npffr2* LV-shRNA and result of CGRP ELISA was analyzed by unpaired Student’s *t-*test. Mice behaviors were analyzed by unpaired Student’s *t-*test or two-way ANOVA with Bonferroni’s multiple comparison test. Immunofluorescence signal was analyzed by two-way ANOVA with Bonferroni’s multiple comparison test. *P*-values below 0.05 were considered statistically significant.

## Results

**Fig. 1:**
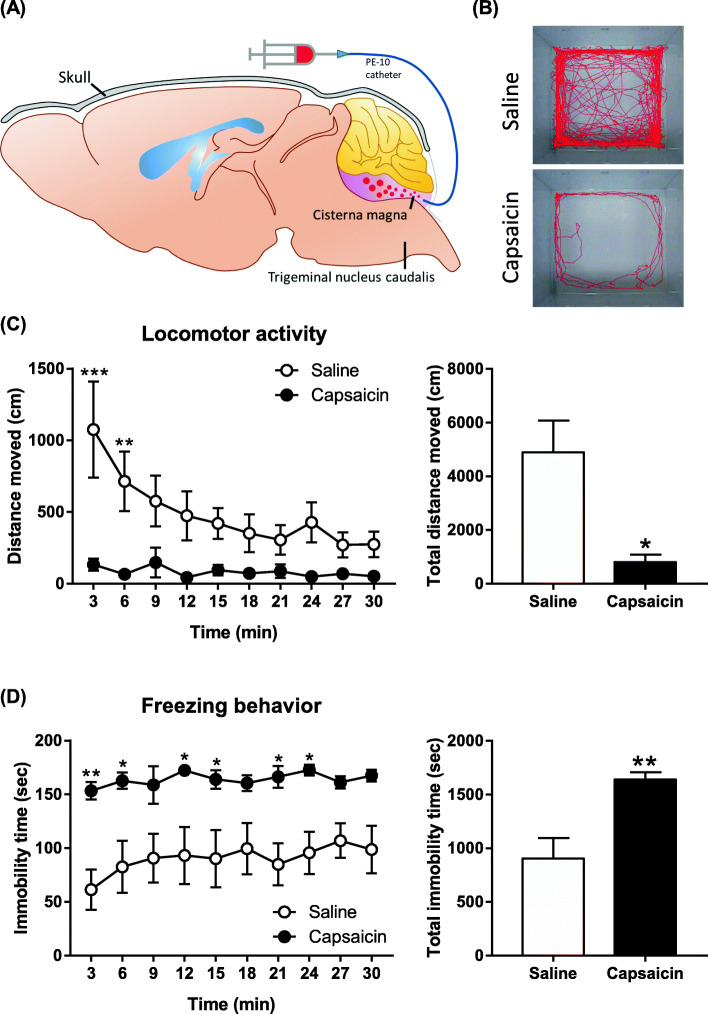
Intracisternal capsaicin injection-induced spontaneous headache-related pain behaviors. Mice were injected with capsaicin into the cisterna magna to induce headache symptoms. The spontaneous headache-related pain behaviors, locomotor activity and freezing behavior, were evaluated 90 min after capsaicin infusion. **a** The illustration of injection site in the cisterna magna. **b** The trace of body movement after saline or capsaicin injection. **c** The locomotor activity was calculated by the distance that mice moved (cm) in every 3 min for a total session of 30 min. Bar graph illustrates cumulated counts within 30 min. **d** The freezing behavior was calculated by the mice immobility time (sec) in every 3 min for a total session of 30 min. Bar graph illustrates cumulated counts within 30 min. Data are represented as mean ± SEM and were analyzed by two-way ANOVA with Bonferroni’s multiple comparison test or unpaired Student’s t-test. *p < 0.05, **p < 0.01, ***p < 0.001, comparing capsaicin and saline treated groups (*N* = 5 per group)

Capsaicin was delivered into the cisterna magna to activate the trigeminovascular pathway and intent to induce the headache symptoms in experimental mice (Fig. 1a illustrates the injection site of cisterna magna). The cisterna magna is close to trigeminal nucleus caudalis and the infused capsaicin can be delivered to trigeminal ganglion or other brain area through cerebrospinal fluid. After the injection of capsaicin for 90 min, the spontaneous headache-related pain behaviors [8] were measured, i.e. locomotor activity and freezing behavior. The trace of body movement was notably decreased after the administration of capsaicin compared to saline control (Fig. 1b). The amount of total movement during 30 min testing period was significantly reduced after capsaicin injection (Fig. 1c). Two-way ANOVA indicates significant effects of capsaicin treatment (F (1, 8) = 11.23, *p* = 0.0101), time (F(9, 72) = 4.087, *p* = 0.0003) and interaction (F(9, 72) = 3.004, *p* = 0.0043). Total movement was significantly reduced after capsaicin injection (*p* = 0.0101). The increased freezing behavior was also noticed (Fig. 1d). Two-way ANOVA indicates significant effect of capsaicin treatment (F (1, 8) = 13.06, *p* = 0.0068). Total freezing time of mice was significantly enhanced after capsaicin injection (*p* = 0.0068).

**Fig. 2:**
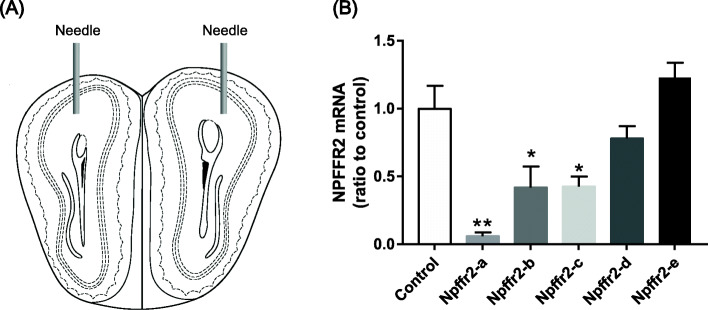
The effect of *Npffr2* gene silencing in the olfactory bulb of WT mice. **a** The illustration of the injection sites in bilateral olfactory bulb. **b** The *Npffr2* gene was silenced by *Npffr2* LV-shRNA. Mice were injected in the olfactory bulb with one of five different *Npffr2* LV-shRNAs (from *Npffr2*-a to *Npffr2*-e). After one week, olfactory bulb tissues were collected to measure *Npffr2* mRNA. Data are represented as the mean ± SEM and were analyzed by unpaired Student’s *t*-test. **p < 0.01; *p < 0.05, compared to the control (*N* = 4 per group)

**Fig. 3:**
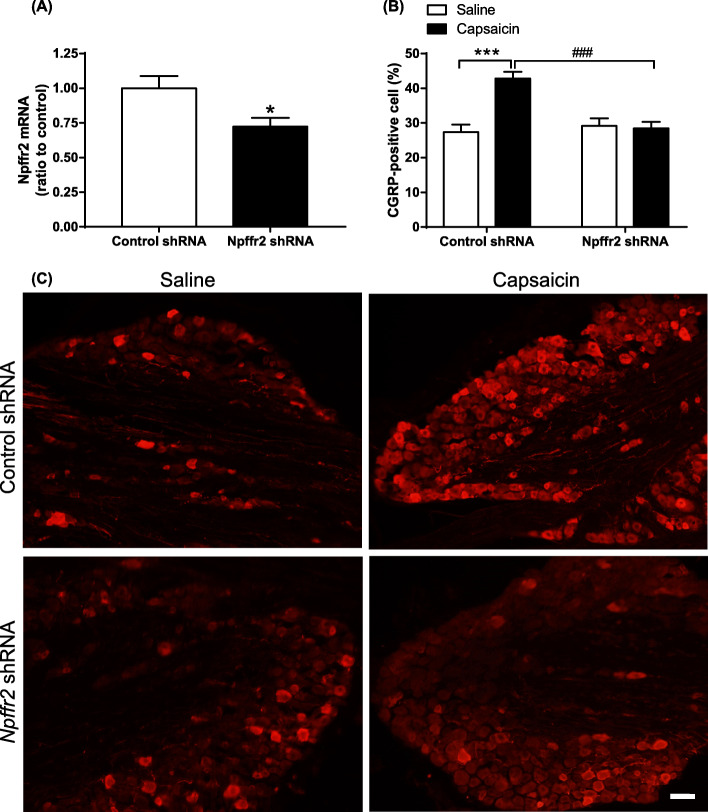
*Npffr2* gene silencing prevents capsaicin-induced CGRP expression in the trigeminal ganglion of mice. One week after mice were intracisternally infused with *Npffr2*-a or control LV-shRNA, the animals were treated with capsaicin for 2 h to activate the trigeminovascular pathway. **a** Trigeminal *Npffr2* gene silencing was measured by real-time PCR one week after LV-shRNA infusion. Data are represented as the mean ± SEM and were analyzed by unpaired Student’s *t*-test. *p < 0.05 compared to the control (*N* = 4 per group). **b** The CGRP protein level in the trigeminal ganglion were measured by immunofluorescence staining. The results are presented as percentage of CGRP-positive cell number. Data are represented as mean ± SEM and were analyzed by two-way ANOVA with Bonferroni’s multiple comparison test. ***p < 0.001, comparing capsaicin and saline treated groups. ^###^*p* < 0.001, comparing control and *Npffr2* LV-shRNA-treated groups (*N* = 5 per group). **c** Immunofluorescence staining of CGRP protein in LV-shRNA- and capsaicin-injected mice. Scale bar = 50 μm

Gene silencing by five LV-shRNA constructs (*Npffr2*-a-e) targeting different sequences in *Npffr2* was tested in WT mice. Olfactory bulb was chosen due to its high expression of NPFFR2 (Fig. 2a illustrates the injection sites). The *Npffr2* LV-shRNAs and control LV-shRNA were injected into the olfactory bulb, and tissues were collected for real-time PCR analysis one week later. *Npffr2*-a (*p* = 0.0015), *Npffr2*-b (*p* = 0.044) and *Npffr2*-c (*p* = 0.020) LV-shRNAs all diminished *Npffr2* mRNA (Fig. 2b). *Npffr2*-a was chosen for further experiments because it exhibited the best silencing rate (94.4 ± 2.9% reduction). Consequently, *Npffr2-*a or control LV-shRNA were injected intracisternally one week before capsaicin treatment. The trigeminal *Npffr2* gene was significantly knockdown a week after the *Npffr2* LV-shRNA infusion (Fig. 3a, *p* = 0.0433). CGRP in the trigeminal ganglion was then evaluated by immunofluorescence staining. Capsaicin upregulated CGRP in the trigeminal ganglion of control LV-shRNA-treated mice. However, *Npffr2*-a LV-shRNA pretreatment attenuated CGRP upregulation (Fig. 3b-c). Two-way ANOVA indicates significant effects of shRNA treatment (F(1, 16) = 9.27, *p* = 0.0077), capsaicin treatment (F(1, 16) = 12.69, *p* = 0.0026) and interaction (F(1, 16) = 15.35, *p* = 0.0012). Bonferroni’s multiple comparison test reveals that the CGRP levels were increased in capsaicin-treated animals compared to saline-treated animals in the control LV-shRNA group (*p* = 0.0001), but the CGRP levels were not increased in the *Npffr2*-a LV-shRNA group. Additionally, CGRP levels were reduced in *Npffr2*-a LV-shRNA-treated animals compared to control LV-shRNA-treated mice after capsaicin injection (*p* = 0.0003).

**Fig. 4:**
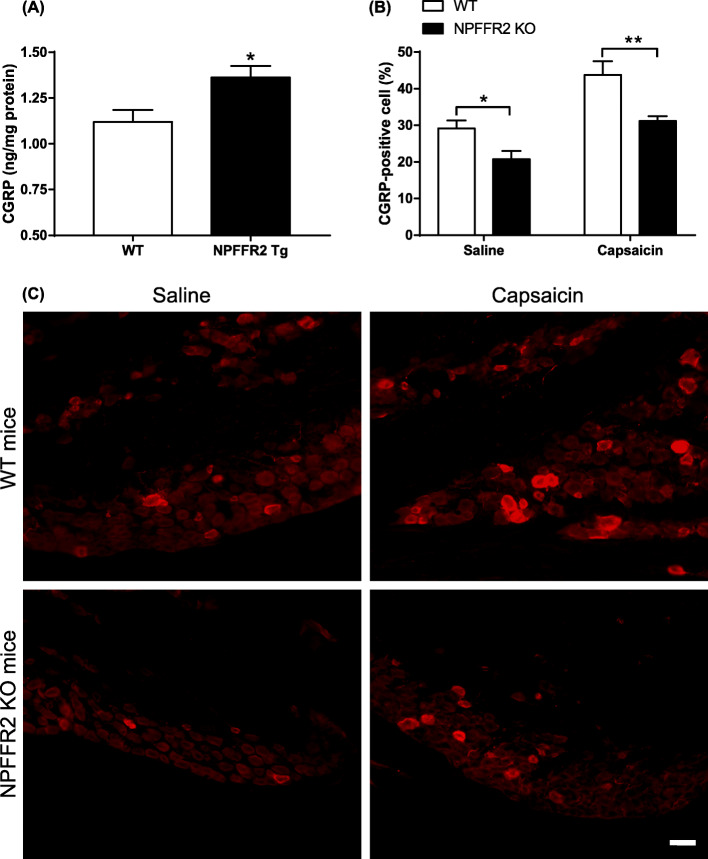
The trigeminal CGRP of NPFFR2 overexpressed Tg mice and the capsaicin-upregulated CGRP expression in NPFFR2 KO mice. **a** The level of CGRP in trigeminal ganglion of NPFFR2 Tg mice was measured by ELISA. Data are represented as the mean ± SEM and were analyzed by unpaired Student’s *t*-test. *p < 0.05 compared to the control (*N* = 9 per group). **b** WT and NPFFR2 knockout (KO) mice were intracisternally infused with capsaicin for 2 h, after which trigeminal ganglion was collected. CGRP protein level in the trigeminal ganglion was measured by immunofluorescence staining. The results are presented as percentage of CGRP-positive cell number. Data are represented as mean ± SEM and were analyzed by two-way ANOVA with Bonferroni’s multiple comparisons test. *p < 0.05, **p < 0.01, comparing WT and NPFFR2 KO mice (N = 5 in saline group, N = 4 in capsaicin group). **c** Immunofluorescence staining of CGRP in WT and NPFFR2 KO mice. Scale bar = 50 μm

The level of CGRP in trigeminal ganglion of NPFFR2 overexpressed Tg mice was measured by ELISA. The expression of CGRP was upregulated in NPFFR2 Tg mice when compared with WT mice (Fig. 4a, *p* = 0.0157). Alternatively, capsaicin was also used to activate the trigeminovascular pathway in NPFFR2 KO mice (Fig. 4b-c). The CGRP level was reduced in NPFFR2 KO mice compared to WT mice in both saline- or capsaicin-treated animals. Two-way ANOVA indicates a significant effect of genotype (F [1, 14] = 18.01, *p* = 0.0008) and capsaicin treatment (F [1, 14] = 25.52, *p* = 0.0002). Bonferroni’s multiple comparison test reveals that CGRP levels were different between the WT and NPFFR2 KO mice in saline (*p* = 0.0464) and capsaicin groups (*p* = 0.0084).

## Discussion

In this study, we evaluated the activation of trigeminovascular pathway induced by intracisternal infusion of capsaicin via measuring CGRP staining in the trigeminal ganglion. Mice pre-treated with *Npffr2* LV-shRNA or NPFFR2 KO mice had reduced capsaicin-upregulated CGRP expression. On the contrary, the CGRP protein was upregulated in the NPFFR2 overexpressed mice. Thus, NPFFR2 seems to play an essential role in initiating the headache by promoting CGRP upregulation in the trigeminal ganglion.

As a neurogenic inflammatory headache model, meningeal stimulation with capsaicin activates trigeminovascular system [6, 7]. Intracisternal capsaicin infusion was demonstrated to activate the trigeminal nucleus caudials (Sp5C) by increasing the *c-fos* positive neurons [9, 10]. The neuronal activation can be inhibited by the traditional migraine medication, Triptans (serotonin 5-HT_1B_/5-HT_1D_ receptor agonist) [9]. Animal models of headache and migraine would exhibit spontaneous pain behaviors, including the decrease of exploration and locomotor activity, or the increase of resting and freezing duration [8]. We demonstrated that intracisternal capsaicin infusion in mice predominately reduced the locomotor activity and increased the freezing behavior during an open field test. The reduction in motor activity was also viewed as a sign of photophobia, the non-pain-related migrainous symptom [6].

Similar to the current findings, meningeal capsaicin stimulation also induced CGRP upregulation in the trigeminal ganglion and peripherally depletion from dura mater [10]. CGRP is an important factor for migraine therapies and diagnosis [2, 11]. Circulating CGRP is increased in migraine patients and after stimulation of the trigeminal ganglion [11, 12]. Although CGRP and substance P are both released after stimulation of the trigeminal ganglion, only CGRP is significantly elevated during acute migraine attacks [13]. Moreover, infusion of CGRP can trigger migraine attacks [3]. The migraine medication, Triptans, inhibits CGRP release, however, its vasoconstrictive effects limit its clinical application [1, 2]. On the other hand, new CGRP-targeting treatments such as anti-CGRP antibodies (Galcanezumab, Fremanezumab and Eptinezumab) and anti-CGRP receptor antibody (Erenumab) have recently reached the market as preventive therapies [2]. Recently (December 23, 2019), the U.S. FDA approved the use of ubrogepant (a CGRP antagonist) for the acute treatment of migraine [14]. Here, our findings indicated that NPFFR2 can regulate the expression of CGRP in the central sensory system, which highlights an unexplored role of NPFFR2 on the development of migraine episode.

NPFF was identified as a risk factor for migraine in patients with and without aura [4]. Its receptor, NPFFR2, plays an important role in the modulation of nociception, and stimulation of NPFFR2 or NPFFR2 overexpression Tg mice increases CGRP level in dorsal root ganglion, leading to peripheral sensitization and hyperalgesia in mice [5]. Furthermore, the NPFFR2 agonist triggers CGRP release from the cultured sensory neurons [5]. Similarly, level of CGRP was upregulated in trigeminal ganglion of NPFFR2 Tg mice. By adopting a capsaicin-activated trigeminovascular pathway conducted in mice, NPFFR2 knockdown/knockout prevents upregulation of CGRP in trigeminal ganglion. Since CGRP is central for triggering headache symptoms [3], our finding suggests that subsequent headache symptoms should also be attenuated.

Silencing effects of five different *Npffr2* LV-shRNA were tested on olfactory bulb due to its high expression of NPFFR2 [15], and this specialized structure makes it easy to be dissected from mice brain. On the other hand, trigeminal nucleus caudalis cannot be clearly dissected without contaminating with other nerve tissues. Because of a highest silencing rate, *Npffr2*-a LV-shRNA was chosen for the designated experiments (94.4% of reduction). The *Npffr2* gene knockdown was then exanimated in the trigeminal ganglion because CGRP was synthesized in the sensory neuron, and the stimulation of NPFFR2 has been previously demonstrated to regulate the CGRP protein synthesis in the dorsal root ganglion [5]. The silencing effect in the trigeminal ganglion (27.67% of reduction) was observed in N*pffr2*-a LV-shRNA-injected mice with a lower silencing rate compared with the effect on olfactory bulb. This could be resulted from a large dilution of LV-shRNA into the cerebrospinal fluid. However, we can still observe a clearly functional impact of *Npffr2* gene knockdown on trigeminal CGRP protein synthesis.

NPFFR2 may be of interest as a target for pharmaceuticals to treat inflammatory pain and cephalic pain. Although several groups are working to develop NPFF receptor antagonists, selective compound(s) targeting NPFFR2 are still lacking. Our data show an important role for NPFFR2 in CGRP-evoked trigeminovascular activation, suggesting that NPFFR2-targeting therapeutics for headache may become valuable clinical tools.

## Data Availability

The datasets used and/or analyzed during the current study are available from the corresponding author on reasonable request.

## Abbreviations

CGRP: Calcitonin-gene-related peptide
NPFF: Neuropeptide FF
NPFFR2: Neuropeptide FF receptor type 2
KO: Knockout
Tg: Transgene
WT: Wild-type
IACUC: Institutional animal care and use committee
LV: Lentivirus
SEM: Standard error mean
