# Correction to: The knockdown of Neuropeptide FF receptor 2 inhibits capsaicin-induced CGRP Upregulation in mouse trigeminal ganglion

**DOI:** 10.1186/s10194-020-01209-z

**Published:** 2020-12-07

**Authors:** Ya-Tin Lin, Zachary Yu, Sze-Chi Tsai, Po-Hung Hsu, Jin-Chung Chen

**Affiliations:** 1 Department of Physiology and Pharmacology, Graduate Institute of Biomedical Sciences, Chang Gung University, 259 Wenhua 1st Road, Guishan Dist, Taoyuan City, 33302 Taiwan; 2 Healthy Aging Research Center, Chang Gung University, 259 Wenhua 1st Road, Guishan Dist, Taoyuan City, 33302 Taiwan; 3 Department of Medicine, Chang Gung University, 259 Wenhua 1st Road, Guishan Dist, Taoyuan City, 33302 Taiwan; 4 Department of Biomedical Sciences, Chang Gung University, 259 Wenhua 1st Road, Guishan Dist, Taoyuan City, 33302 Taiwan; 5 Center for Advanced Molecular Imaging and Translation, Chang Gung Memorial Hospital, 5 Fu-Hsing Street, Guishan Dist, Taoyuan City, 33302 Taiwan; 6 Department of Electrical Engineering, Chang Gung University, 259 Wenhua 1st Road, Guishan Dist, Taoyuan City, 33302 Taiwan; 7 Neuroscience Research Center, Chang Gung Memorial Hospital, 5 Fu-Hsing Street, Guishan Dist, Taoyuan City, 33302 Taiwan

**Correction to: J Headache Pain 21, 87 (2020)**

**https://doi.org/10.1186/s10194-020-01152-z**

After publication of the original article [1], we were notified of a mistake in the article title. The authors have found that mice knockdown/ or knockout of NPFFR2 showed no responses to capsaicin injection, which up-regulated the CGRP protein in the trigeminal ganglion. Therefore, the word “knockdown” should be added into the title to correctly represent the paper’s major findings.

The correct title should read:

“The knockdown of Neuropeptide FF receptor 2 inhibits capsaicin-induced CGRP Upregulation in mouse trigeminal ganglion”.
